# Physic and Music

**Published:** 1855-02

**Authors:** 


Physic and Music.—Every one knows that Esculapius was the son of Apollo, and this relationship accounts for the frequency with which we find good physicians to be good musicians also. Our annals are rich in distinguished doctors, who have had excellent voices, or who played with skill on various instruments. Boerhaave was a capital performer on the flute ; and it is a curious ccincidence that the late great texicologist of France, and the eminent living texicologist of Great Britain, should have both possessed splendid bass voices. The editor of the *Union Medicale* comments on the number of musical confreres he meets with in the saloons of Paris (Janvier 14) ; and we can assure him that in Edinburgh the same thing is observable. In leed, most of our public professional dinners and entertainments would now be considered dull affairs, without the songs and glees with which they are enlivened by the Medical Glee Club of this city.—*Ed. Med. Journal.*

